# A Method for Tracking the Time Evolution of Steady-State Evoked Potentials

**DOI:** 10.3791/59898

**Published:** 2019-05-25

**Authors:** Pavel Prado-Gutiérrez, Mónica Otero, Eduardo Martínez-Montes, Alejandro Weinstein, María-José Escobar, Wael El-Deredy, Matías Zañartu

**Affiliations:** 1 Advanced Center for Electrical and Electronic Engineering (AC3E), Universidad Técnica Federico Santa María; 2 Department of Electronic Engineering, Universidad Técnica Federico Santa María; 3 Neuroinformatics Department, Cuban Neuroscience Center; 4 Centro de Investigación y Desarrollo en IngenierÍa, Universidad de Valparaíso

**Keywords:** Neuroscience, Issue 147, adaptation, auditory steady-state responses, averaging method, entrainment, habituation, noise cancelation, neural dynamics, steady-state-visually evoked potentials

## Abstract

Neural entrainment refers to the synchronization of neural activity to the periodicity of sensory stimuli. This synchronization defines the generation of steady-state evoked responses (i.e., oscillations in the electroencephalogram phase-locked to the driving stimuli). The classic interpretation of the amplitude of the steady-state evoked responses assumes a stereotypical time-invariant neural response plus random background fluctuations, such that averaging over repeated presentations of the stimulus recovers the stereotypical response. This approach ignores the dynamics of the steady-state, as in the case of the adaptation elicited by prolonged exposures to the stimulus. To analyze the dynamics of steady-state responses, it can be assumed that the time evolution of the response amplitude is the same in different stimulation runs separated by sufficiently long breaks. Based on this assumption, a method to characterize the time evolution of steady-state responses is presented. A sufficiently large number of recordings are acquired in response to the same experimental condition. Experimental runs (recordings) are column-wise averaged (i.e., runs are averaged but epoch within recordings are not averaged with the preceding segments). The column-wise averaging allows analysis of steady-state responses in recordings with remarkably high signal-to-noise ratios. Therefore, the averaged signal provides an accurate representation of the time evolution of the steady-state response, which can be analyzed in both the time and frequency domains. In this study, a detailed description of the method is provided, using steady-state visually evoked potentials as an example of a response. Advantages and caveats are evaluated based on a comparison with single-trial methods designed to analyze neural entrainment.

## Introduction

When recorded from the scalp, brain electrical activity is observed as continuous and regular changes in voltages over time. This electrical activity is called electroencephalogram (EEG) and was first described by Hans Berger in the late twenties of the last century^1^. Subsequent seminal studies described the EEG as a compound time series, in which different rhythmic or repetitive patterns can be observed^2,3,4^. Nowadays, the EEG is typically divided into five well-established frequency bands, delta, theta, alpha, beta, and gamma, which are associated with the different sensory and cognitive process.

For years, the study of brain oscillations using EEG was restricted to either analysis of the spectrum in the ongoing activity or changes in oscillatory activity elicited by non-periodic sensory events. In the last decades, different methodologies have been implemented for modulating ongoing EEG oscillations and exploring the effects of such modulations on perceptual and cognitive processes, including the presentation of rhythmic sensory stimulation for inducing neural entrainment. The term neural entrainment refers to the synchronization of neural activity with the periodic properties of sensory stimuli. This process leads to the generation of steady-state evoked potentials (i.e., EEG oscillations locked to the periodic properties of the driving stimuli). Steady-state evoked potentials are most commonly elicited by visual, auditory, and vibrotactile stimulation, using either transient stimuli presented at a constant rate or continuous stimulation modulated in amplitude at the frequency of interest. Whereas somatosensory steady-state evoked potentials (SSSEPs) are recorded in response to repetitive tactile stimulation^5,6^, steady-state visually evoked potentials (SSVEPs) are generally elicited by the periodic presentation of luminance flickers, pictures, and faces^7,8^. Auditory steady-state responses (ASSRs) are usually generated by trains of transient acoustic stimuli or by the continuous presentation of amplitude-modulated tones^9,10^.

The extraction of steady-state evoked potentials from the measured EEG essentially relies on averaging subsequently acquired EEG epochs time-locked to the stimulus^11^. Due to the periodicity of the responses, they can be analyzed in both time and frequency domains. After the frequency-domain transformation, the sensory response is observed as peaks of amplitude at the presentation rate or modulation frequency of the external stimuli, and their corresponding harmonics. These procedures (time-domain averaging and the subsequent frequency-domain transformation) have been essential for developing a hearing test based on the detection of ASSR methods with clinical purposes^12,13,14,15,16^.

Furthermore, the classical time-domain averaging of EEG epochs has been extremely useful for analyzing physiological processes such as the generation and extinction of SSVEP^17,18^. Presenting consecutive trains of flicker lights and averaging subsequent epochs within a recording, Wacker et al.^19^ observed that the phase-locking index of the SSVEP rapidly increased during the first 400 ms of stimulation and remained high afterwards. They also reported that robust visual entrainment was established between 700–1 100 ms after stimulus onset. A certain degree of entrainment remained effective after the offset of the stimulation train, which lasted approximately three periods of the oscillatory response^17,19^. Those behaviors have been interpreted as the engaging/disengaging effect of the observed oscillations, which is a consequence of the nonlinear information processing in the human visual system^17^. Alternatively, it is known that under certain experimental conditions, the flicker stimulation can elicit on-responses at the beginning, and off-responses at the end of stimulation trains instead of neural entrainment^18^.

The main assumption to average consecutively acquired EEG epochs is that the EEG signal represents a linear combination of the sensory response and the background noise^20^. Furthermore, the amplitude, frequency, and phase of the oscillatory response are assumed to be stationary, whereas the background noise is considered as a random activity. However, in cases in which this assumption is not met, the response amplitude computed after several epochs do not necessarily correspond to the instantaneous amplitude of the evoked potential.

It has been recently reported that the ASSR generated in the brainstem of rats adapts to the continuous presentation of amplitude-modulated tones (i.e., the response amplitude decrease exponentially over time)^21,22^. Adaptation has been interpreted as a neural mechanism that reflects the loss of novelty of a monotonously repetitive sensory stimulus, increasing the sensitivity to relevant fluctuations in the acoustic environment^23,24^. In the auditory pathway, adaptation may enhance speech comprehension in noisy environments. Furthermore, this process may be a part of existing mechanisms to monitor the auditory feedback of one’s own voice to control the speech production.

Analyzing the time evolution of the 40 Hz ASSR in humans, Van Eeckhoutte et al.^25^ observed a significant but small decrease in the response amplitude over time (around −0.0002 μV/s based on the group analysis, when assuming a linear decrease over time). Consequently, these authors concluded that the 40 Hz ASSR in humans does not adapt to the stimulation. In humans, non-stationary behaviors have been observed when analyzing the stability of the SSVEP^26^. These authors observed that the amplitude of the fundamental frequency and the second harmonic of the SSVEP were stationary in only 30% and 66.7% of the subjects they tested, respectively. The phases of both SSVEP frequency components, although relatively stable over time, exhibited small drifts^26^.

Therefore, although the classical time-domain averaging of subsequently acquired epochs allows exploring of stationary properties of the neural entrainment, this methodology needs to be revised when long-term dynamics of the entrainment is the focus of the research, or when the averaging of short-term dynamics is corrupted by the occurrence of long-term dynamics. To characterize non-stationary behaviors of the steady-state responses, the evoked response computed at a given time window should not be compromised by those computed in the preceding EEG segments. In other words, the evoked potential should be extracted from the background noise without epochs being time-domain averaged with the preceding EEG segments.

In this study, a method for assessing the dynamics of neural entrainment is presented. Steady-state responses are repetitively recorded in response to the same stimulation, where consecutive recordings are interleaved by a resting interval of three times the length of the experimental run. Considering that if the time evolution of the physiological response is the same in different independent experimental runs (independent recordings), recordings are column-wise averaged. In other words, epochs corresponding to the same location in the different recordings are averaged, without averaging epochs within a recording. Consequently, the response amplitude computed at any stimulation interval will correspond to the instantaneous amplitude of the evoked potential. The sensory responses can be either analyzed in the time-domain or transformed into the frequency-domain, depending on the aim of the experiment. In any case, the amplitudes can be plotted as a function of time to analyze time evolution of the steady-state response. Generation and extinction of the steady-state evoked potentials can be assessed by restricting the analysis to the first and last epochs of the recordings.

The dynamics of the neural entrainment can be analyzed using other approaches, such as narrowband filtering single-trial measurements around the frequency of interest and computing the envelope of the power signal using low-pass filtering^25^ and the Hilbert transformation^27^. Compared to these methodologies, the column-wise averaging of epochs allows computing steady-state parameters based on signals with the higher signal-to-noise ratio (SNR). Recently, Kalman filtering has emerged as a promising technique for the estimation of 40-Hz ASSR amplitudes^28,29,30^. Implementation of Kalman filtering can improve the detection of steady-state responses closer to the electrophysiological threshold and reduce the time of the hearing test^29^. Furthermore, stationary responses are not needed to be assumed when a Kalman filtering approach is used to estimate the ASSR amplitude^30^. Nevertheless, only one study has analyzed the time evolution of ASSRs using Kalman filtering^25^. The conclusion of the study is that the 40-Hz ASSR amplitude is stable over the stimulation interval. Therefore, Kalman filtering needs to be tested in conditions under which the ASRR is not stationary.

Although time consuming, the column-wise averaging method is model-free and does not need initialization values and/or a priori definitions of the noise behavior. Furthermore, since it does not involve convergence times, the column-wise averaging may provide a more reliable representation of the onset of neural entrainment. Therefore, the results obtained with the column-wise averaging method can be considered as the ground truth for analyzing dynamics of the neural entrainment using Kalman filtering.

This description of the protocol is based on an example of SSVEP. However, it is important to note that the method presented here is modality-independent, such that it can also be used to analyze the time evolution of SSSEP and ASSR.

## Protocol

The present study was performed under approval of the Research and Ethics Committee of the Universidad de Valparaíso, Chile (assessment statement code CEC170–18), confirmed to the national guidelines for research with human subjects.

### 1. Preparation

1. Welcome the subject.
2. Explain the aims and relevance of the study. Provide a description of relevant technical details. Answer all questions thoroughly.
3. Explicitly mention that she/he can interrupt the experimental session at any time if desired.
4. Ask the volunteer to read the Subject Informed Consent and sign the corresponding form. Interrupt the experimental session if the informed consent is not obtained.

### 2. Subject preparation

1. Ask the subject to sit in a laboratory chair in a comfortable position.
  1. Clean the scalp with ethanol (a solution at 95%) to remove the layer of dead skin cells and sebum that cover it. This step is important to reduce impedance between the electrodes and scalp.
2. Measure the head circumference with a measuring tape to define the size of the electrode cap to be used.
  1. Ask the subject to wear the electrode cap. Provide the instructions for comfortable but correct positioning of the cap.
  2. Measure the distance between the nasion (Nz, the middle point of the nasofrontal suture, which can be identified by the depression between the eyes and the top of the nose) and the inion (Iz, the prominence of the occipital bone) using a measuring tape.
  3. Measure the distance between the left and right pre-auricular points (identified as the depression just before the auricle of the ears) using a measuring tape.
  4. Correct the position of the electrode cap, so the intersection between the imaginary lines defined in the previous steps correspond to the vertex of the head. Make sure the subject is comfortable after the adjustments.
3. Put conductive gel in the electrode holders, according to the locations considered for the experiment.
  1. Use 64 scalp locations following the International 10–20 system^31^ to use the outcome of the protocol to perform source localization analysis. The higher number of the electrode locations (128) on the scalp can be used if needed.
  2. Implement clinical or ambulatory settings (with only a few electrodes) if source localization analysis is not planned. Use occipital locations to record SSVEP, temporal locations to acquire ASSR, and parietal locations to record SSSEP.
  3. Push the electrodes in the electrode holders. Make sure that the label of the electrode matches the label of location in the cap.
  4. Accompany the volunteer to the experimental room (preferably, a shielded, sound-attenuated chamber). Ask the subject to sit in a chair inside the room, in a comfortable position.
4. Place external electrodes on the nose and earlobes if a physical reference (different from the scalp electrodes) will be used for re-reference the EEG recording (in step 3.8.1).
5. Place external electrodes in periocular locations.
  1. Place electrodes on the cheek and the frontal region of the head, approximately 1 cm above the eyebrow, to record blinking (in step 2.6.1).
  2. Place electrodes on the outer canthus of the eyes, approximately 1 cm above/below the midline, to record eye movements (in step 2.6.1).
    NOTE: The electrooculogram (EOG) will be used in the step 3.8.5 for removing EGG artifacts induced by blinking and eye movements.
6. Turn the EEG acquisition system on and check the electrode impedance if a low-impedance system is used for recording the EEG. Correct the impedance, as needed, as per the manufacturer’s directions. Impedance should be kept below 10 kΩ^32.^
  1. Ask the subject to blink and move the eyes in different directions to ensure that EOG is being correctly recorded.
7. To analyze the dynamic of SSVEP, adjust the location of the screen in the vertical direction, to match the view angle of the subject. Dim the lights of the room until a comfortable level is achieved. Adjust the luminance level of the screen to the upper limit of the participant’s comfort level.
  1. To analyze the dynamics of ASSR, insert the earphones using the correct foam inserts, so the earphones fit the ear canal. Check that sounds are delivered at the desired intensity (e.g., a psychophysical comfortable level^33^).

### 3. EEG acquisition and pre-processing

1. Set the stimulus parameters defined in the experimental design. Refer to the user manual provided by the manufacturer of the stimulation system for details about the software.
  NOTE: For comprehensive explanations of the stimulus used for generation of SSVEP and ASSR, see Norcia et al.^8^ and Rance^34^, respectively.
2. Instruct the subject to pay attention to the stimulation, in the case that visual entrainment is the topic of the experiment.
  1. Present a subtitled movie with the sound off when auditory entrainment is the topic of the experiment.
    NOTE: Presentation of a silent movie allows deflection of attention from the acoustic stimulation while maintaining arousal level^25^.
3. Present stimuli longer than 90 s, as have been done to investigate the time evolution of SSVEPs and ASSR in both humans and animal models^21,22,25,26^.
  NOTE: Present stimuli shorter in duration if a pilot study has been performed.
4. Pause the stimulation for 2 min if only one experimental condition is being tested. Interact with the subject to check awareness.
  NOTE: The duration of the pause depends on the duration of the stimulation. Pauses 3x longer than the stimulation intervals will ensure that a response elicited by one stimulus is not affected by the previous stimulation. Longer pauses are permitted if the subject so requests.
  1. Pause the stimulation for at least 10 s when different experimental conditions are tested since alternating stimulation with pauses of 10 s has been proposed to decrease extra adaptation effects and reduce the length of the experiment^25^.
5. Repeat the presentation steps (steps 3.3–3.4) at least 30x to ensure the high SNR of the measurements after the averaging of epochs (step 4.4).
6. Record the EEG using standard procedures^35^. Create a separate EEG file for each experimental run. NOTE: Refer to the user manual of the acquisition system for details about the software.
7. Monitor the EEG recording to detect sleep periods based on the level of alpha activity and the frequency at which blinking artifacts appear. Pause the experiment when increased alpha levels accompanied by decreased blinking frequencies are detected, which is indicative of sleepiness. Reject the experimental run from further analysis when sleep periods are detected.
  1. Compute the amplitude of the steady-state response at the end of each experimental run, following the instruction provided in the user manual of the acquisition software used in the experiment.
  2. Monitor the attentional level of the subject by comparing the amplitude of steady-state responses obtained at the end of each experimental run. Set the steady-state amplitude obtained in the first experimental runs as a reference amplitude.
  3. Set a rejection threshold (a decrease in the response amplitude of 5% regarding the reference amplitude). Reject the experimental runs in which the amplitude of the steady-state response meets the rejection criterium.
  4. Finish the experimental session after acquiring the number of runs defined in the experimental design.
8. Pre-process the EEG data offline using standard EEG procedures^35^ described in the next steps per the manufacturer’s directions.
  1. Re-reference the recording using an average reference (average of all recording electrodes) or the average of a subset of electrodes. Alternatively, use a physical reference (e.g., external electrodes placed on the nose and earlobes described in step 2.4).
  2. Convert the electrode coordinates to the international 10/20 system if the radial coordinate system was used during the EEG acquisitions. Refer to the manufacturer manual for details on conversion.
  3. Band-pass filter the EEG signal between 0.5–300 Hz. Set a notch-filter (centered at 50 Hz or 60 Hz) if necessary.
  4. Down-sample the EEG signal to decrease the execution time of the algorithm selected for removing ocular artifacts (step 3.8.5). NOTE: A sampling frequency of 512 Hz is adequate to analyze brain oscillations of frequency below 40 Hz^35^.
  5. Remove the ocular artifacts.
    NOTE: To this end, different techniques can be used (see Urigüen and Garcia-Zapirain^35^ for an extensive review on artifact removal algorithms). Among them, independent component analysis is one of the most extended methodologies and is implemented in both commercial and free analysis softwares^37,38,39^.
  6. Segment the EEG data in epochs time-locked to stimulation. Select the epochs length according to the aim of the experiment.
    NOTE: Epochs should be sufficiently long to allow for analysis of the steady-state response in the frequency-domain with an adequate spectral resolution.
  7. Do not run artifact rejection algorithms at this stage to detect and remove epochs containing artifacts.
    NOTE: Removing epoch at this stage will induce errors when the dataset is organized to run the column-wise averaging of epochs (steps 4.2 and 4.4). Rejection algorithms are implemented at a later processing step (step 4.1.4).
  8. Run the DC-detrend function to calculate DC-trends in individual EEG epochs and correct them.
  9. Run the baseline correction function to correct the baseline of the recording. Select pre-stimulus time intervals longer than 200 ms. NOTE: Baseline correction consists of averaging the data in the selected time interval. The average is calculated for each channel and subtracted from each data point in every epoch.

### 4. Computation of the response amplitudes

**Figure 1:**
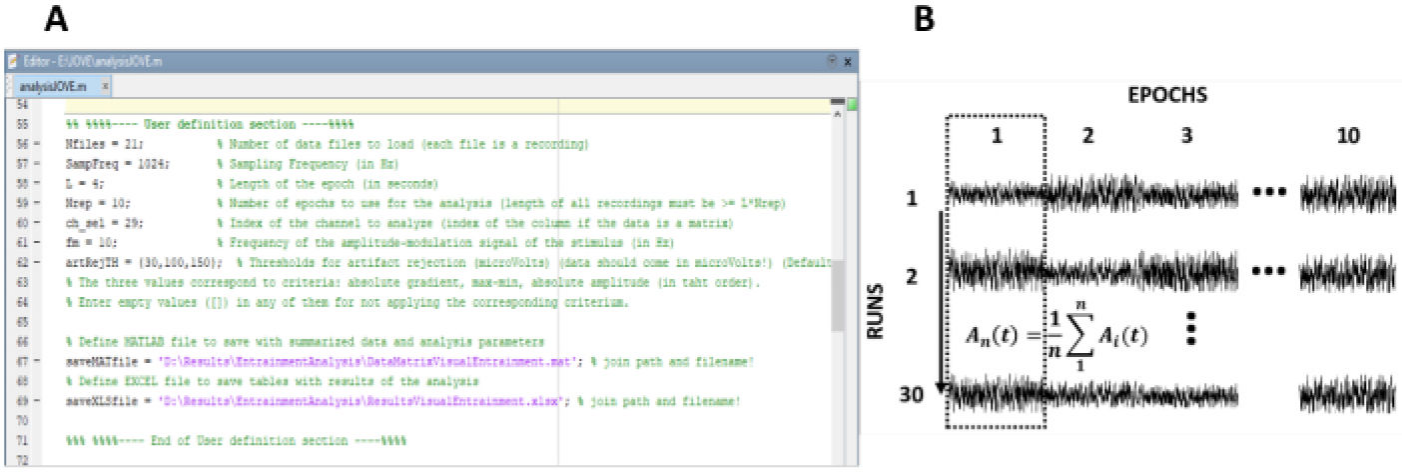
Critical steps for extracting the time evolution of the amplitude of steady-state responses. (**A**) Screenshot of the processing code, where analysis parameters are defined. (**B**) Representative diagram illustrating the organization of the dataset. A data matrix composed of 30 recordings of 10 epochs is represented. The column-wise averaging of epochs is highlighted in the first column. The vertical line represents the direction of the averaging.

1. Enter the parameters needed for the computation of the steady-state responses (Figure 1A).
  NOTE: The in-house code used for processing the data is freely available at <https://figshare.com/projects/Steady-state_visually_evoked_potentials_SSVEP_elicited_in_humans_by_continuos_light_modulated_in_amplitude_at_10Hz/62573>. Refer to the help text inside the code for further instructions. Similarly, a subset of the data used in this study is available.
  1. Enter the number of recordings (experimental runs) of the experiment.
  2. Enter the length of the epochs to segment the individual recordings.
  3. Enter the sampling frequency of the experiment.
  4. Select artifact rejection algorithms to detect and remove epochs containing artifacts. The available selection criteria are 1) gradient (absolute difference between two consecutive samples), 2) max-min (the difference between the maximum and minimum amplitude in the epoch), and 3) amplitude (absolute maximum and minimum amplitudes).
  5. Run the processing code.
    NOTE: Steps 4.2–4.7 are automatically performed when this option is selected. Run the steps manually if appropriate.
2. Re-arrange the epochs into a data matrix of **n** rows and **m** columns, in which **n** represents the number of recordings (experimental runs) and **m** the number of epochs (Figure 1B).
3. Weight the epoch to attenuate the effect of motion and muscular artifacts.
  NOTE: Weighted EEG epochs are obtained by dividing each voltage sample by the amplitude variance of the epoch they belong to, so that variance is used as a measure of amplitude variability and weighting factor^40^.
4. Column-wise average the dataset. To this end, time-domain average the epochs corresponding to the same time window in the different recordings.
  NOTE: This step allows the computation of the steady-state amplitude in recordings with a remarkably high signal-to-noise ratio (SNR).
5. Export the time series resulting from the averaging for further analysis of the time evolution of the entrainment in external software.
  1. Compute the amplitude of the steady-state response in each epoch resulting from the column-wise averaging, using the fast Fourier transform (FFT).
    NOTE: The FFT length should correspond to the length of one epoch. The implementation of a windowing technique is not mandatory. The amplitude of the steady-state response is defined as the spectral amplitude obtained at the frequency of the amplitude modulation of the sensory stimuli.
6. Vector average the amplitude of an ad-hoc number of FFT bins at each side of the frequency of the response to calculate the residual noise level (RNL). The number of FFT bins should correspond with a frequency band of about 3 Hz, at each side of the frequency of the response. NOTE: The high frequency-specificity of the steady-state responses makes the response amplitude independent of those background oscillations with similar frequencies, which in turn distributes uniformly in a relatively narrow frequency band^41,42,43^.
7. Plot the amplitude of the steady-state response and the RNL as a function of column index (i.e., the number of the acquired epoch) to explore the evolution of the steady-state response during the stimulation interval.

## Representative Results

SSVEP was elicited by continuous visual stimuli of 40 s in length, where the light intensity was modulated by a sinusoidal wave of 10 Hz (modulation depth of 90%). Stimuli were delivered by four light-emitting diodes (LEDs) situated in the center of a 50 cm × 50 cm black screen, as vertexes of a 5 cm × 5 cm square. When the participant sat 70 cm from the screen, the area of the square of LEDs subtends a visual angle of about 4°. The LED screen was designed using an USB-based microcontroller development system and four super bright white LEDs of 10 mm of diameter. The pulse width modulation (PWM) technique was used to control the power supplied to the LEDs. This technique controlled the LEDs intensities at a given frequency and generate the final sinusoidal envelope. A PWM frequency of 40 kHz was used to avoid a perceivable flicker effect.

Thirty recordings were obtained, which were segmented in epochs of 4 s. Therefore, a dataset composed of 10 columns (number of EEG epochs within recordings) and 30 rows (number of recordings, number of experimental runs) was obtained.

**Figure 2:**
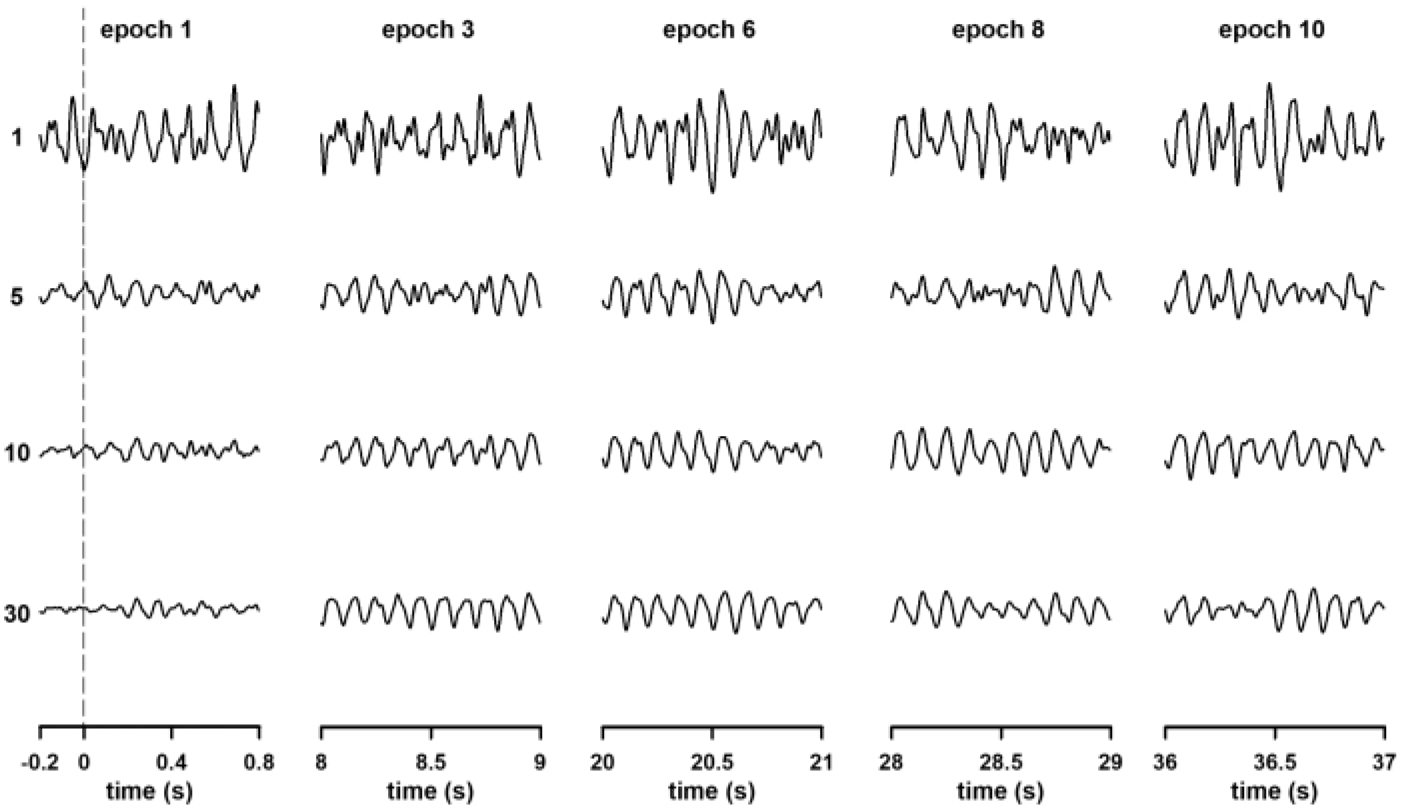
Changes in the waveform of steady-state visually evoked potentials (SSVEP) during the column-wise averaging of epochs. Responses were elicited by the continuous presentation of light modulated in amplitude at 10 Hz. The rows show the waveforms obtained after averaging all previous recordings (i.e., row 1 is the first recording, row 5 is the waveform obtained after averaging the first five recordings, and the last row is the average of all recordings). More reliable waveforms of SSVEP were observed in each column as the number of averaging runs increased. To provide clarity (to make the oscillations of the SSVEP visible), only the first second of the epochs is represented. The exceptions are traces in the first column of the data set, for which 0.2 seconds of pre-stimulus baseline are displayed.

The neural oscillation time-locked to the stimulation became evident as the column-wise averaging was performed (Figure 2). Significantly, the interval at which the SSVEP is generated can be observed in traces corresponding with column 1. In that column, 0.2 s of pre-stimulus baseline are plotted in addition to the first 0.8 s of neural entrainment. Therefore, the procedure described here allows characterization of 1) the dynamics of the oscillatory response once neural entrainment is already established and 2) the engagement of neural oscillations. One or more epochs recorded after the end of stimulation can also be included in the data matrix to study extinction of the steady-state response after stimulus offset.

**Figure 3:**
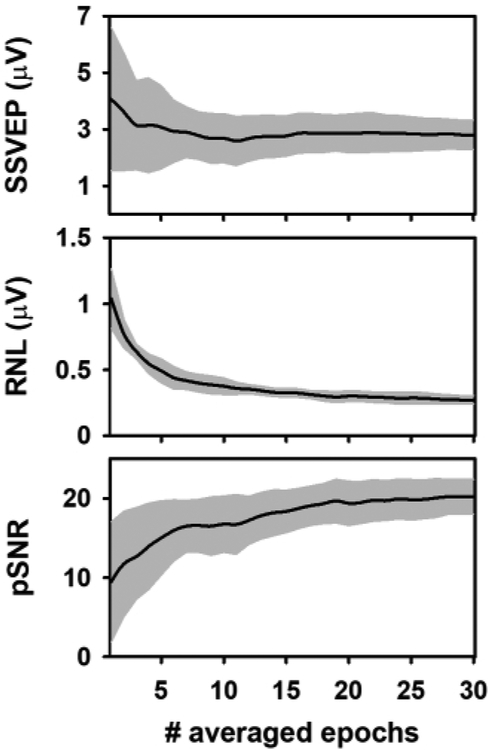
Changes in the response and recording parameters during the column-wise averaging of epochs. (**A**) Evolution of the SSVEP amplitude. **(B**) Behavior of the RNL. (**C**) Changes in the pSNR. Black lines represent the mean values obtained for each column (n = 10) and the grey shadow represents the area covered by ± one standard deviation.

During the column-wise averaging of epochs, the mean amplitude of the SSVEP (spectral amplitude at 10 Hz, computed by applying the FFT) decreased during the averaging of the first epochs of the columns and tended to stabilize afterward (Figure 3A). This result agrees with previous studies analyzing the evolution of ASSR during the averaging of sequentially acquired epochs^21,22,40,43,44^. The behavior of the response amplitude during averaging is usually explained by the relatively high contribution of unaveraged noise to the response amplitude computed in the first epochs, which is attenuated as averaging is performed^13,44,45,46,47^. Noteworthy, the SSVEP amplitude variability significantly decreased as averaging progressed.

We also analyzed the RNL of the measurements during the column-wise averaging of epochs (Figure 3B). The RNL was computed in a narrow frequency band (3 Hz) at both sides of the frequency of the SSVEP. Although this procedure is not common when SSVEP are analyzed, vector-averaging a given number of frequency bins around that of the neural entrainment is the standard for estimating the RNL in ASSR measurements^41,42,43^. As expected, the RNL progressively decreased as the number of averaged epochs increased and reached the asymptotic level after about 20 epochs were processed. Unlike that observed when the SSVEP amplitude was analyzed, the standard deviation of the RNL remained relatively constant as the number of averaged epochs increased, which suggests that the recording conditions were stable along the experimental session.

The results presented above determined the changes in the peak signal-to-noise ratio (pSNR) of measurements during the column-wise averaging of epoch (Figure 3C). This term is defined here as the ratio (in dB) between the square amplitude of the response (SSVEP) and square amplitude of the RNL. As averaging progressed, the pSNR increased as the number of averaged epochs increased up to 18, approximately. Further increments in the number of averaged epochs did not significantly impact the quality of the signal. The variability of the pSNR decreased as more epochs were averaged.

**Figure 4:**
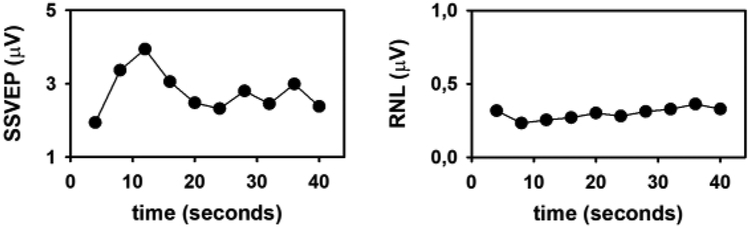
Time evolution of the SSVEP elicited by the presentation of continuous visual stimulation, modulated in amplitude at 10 Hz. (**A**) Time course of the SSVEP amplitude. (**B**) Time course of the RNL.

Finally, the dynamics of the SSVEP amplitude and the RNL are represented in Figure 4. These time evolutions were obtained by plotting the response parameters computed at the end of the column-wise averaging of epochs as a function of the number of columns (as a function of time). As demonstrated by Labecki et al.^26^, the dynamics of SSVEP can significantly vary among subjects. Since the results presented in Figure 4 correspond to a single individual, generalizations cannot be made. In this subject, the amplitude of the SSVEP displayed a relatively complex behavior (Figure 4A). The response amplitude gradually increased during the first 12 seconds following the stimulus onset (time which corresponds to the length of 3 epochs). As the stimulus persisted, the SSVEP consistently decreased during the following 12 seconds, and remained relatively constant afterwards. These results cannot be explained by the behavior of the RNL, since this parameter was relatively constant during the stimulation interval (Figure 4B). The increase in the SSVEP amplitude following the stimulus onset is evident in the traces presented in Figure 2 and can be explained by integration processes, which result in stabilization of the neural entrainment. The subsequent decrease in amplitude suggests the adaptation of SSVEP to the sustained stimulation. Nevertheless, these hypotheses need to be tested in controlled experiments with appropriated sample sizes.

## Discussion

This work describes an experimental procedure for analyzing the dynamics of oscillatory brain responses. Such methodology consists of acquiring a sufficient number of independent experimental runs of the same experimental condition, and time-domain averaging epochs corresponding to the same time window in the different recordings (columns-wise averaging in Figure 1B). The amplitude computed in the averaged data represent the instantaneous amplitude of the oscillatory response. Plotting these amplitudes as a function of time (or the number of columns in the dataset) allows analyzing the time evolution of the oscillatory response time-locked to the stimulation. This methodology is a modification of that proposed by Ritter et al.^23^ for analyzing the adaptation of transient cortical evoked potentials. The method has been used to analyze the dynamic of auditory evoked potentials in both humans^24^ and animal models^20,21^.

From a methodological point of view, the combination of parameters used to elicit the steady-state response and those implemented to extract the neural response from background noise is critical to analyze the time evolution of steady-state evoked potentials^22^. The stimulus length used in the experiment presented here (40 s) was selected based on results obtained in a pilot study. This stimulus length was sufficient to analyze the adaptation of ASSR generated in the rat brainstem^21,22^. Furthermore, the stimulus length should exceed the time at which the asymptotic instantaneous band power of SSVEPs is reached (Figure 1 in Labecki et al.^26^). Nevertheless, the asymptotic instantaneous band power of SSVEPs can be reached beyond 60s in some cases (Figure 2 in Labecki et al.^26^). Therefore, running a small-sample pilot study is recommended to define the stimulus length of the stimulation. Otherwise, a stimulus length longer than 90 s is recommended to achieve complete representation of the time evolution of the response. Using adequately long pauses between consecutive recordings implies considering consecutive experimental runs as to be statistically independent (i.e., different, independent measures of the same variable). To the best of our knowledge, no experiments have been performed to analyze the optimum pause between runs (minimum pause required to make runs independent from each other). The criterium of using pauses at least 3x longer than the stimulus length is conservative enough to ensure that the steady-state response recorded in any given run is not affected by the previous stimulation.

Recently, alternating stimuli (experimental conditions) has been proposed as a choice to reduce the pause between experimental runs, avoiding extra adaptation effect^25^. Likewise, the number of experimental runs (30) implemented in this experimental protocol is conservative, since the asymptotic RNL and pSNR are typically reached after averaging 20 experimental runs, approximately. When stimuli fall within the middle-upper region of the dynamic range of the response (high sensation levels), lower numbers of runs are likely needed to analyze the dynamics of the evoked response. Nevertheless, in cases in which different experimental conditions are tested, having the same number of experimental runs is crucial for making comparisons among conditions (i.e., different sensation levels).

In addition to the column-wise averaging of epochs, the dynamics of oscillatory evoked potentials has been analyzed by filtering the single-trial measurements in a narrow frequency band around the frequency of interest and computing the envelope of the power signal using low-pass filtering^26^. Likewise, single trial analysis has been implemented to characterize the transition period that precedes the stable region of SSVEP^48^, and the changes in amplitude and phase of the SSVEP during the stable region of the response^49^. While single trial analyses allow discrimination of relatively fast fluctuations in response amplitude, experimental designs to analyze the average response in blocks separated with a given inter-block interval only account for long-term variations in the amplitude of the evoked potential^50,51^. The column-wise averaging of epochs stands between these two options. Converting the averaged signal to the frequency-domain using the FFT implies analyzing the dynamics of the response with a resolution equal to the length of the epoch. In the example presented here, the SSVEP was reported every 4 s. Although 4 s of resolution is adequate to describe dynamics occurring at intervals of time surpassing tens of seconds, such as that of the SSVEP^26^, partially overlapping epochs in the original recordings allows to describe the time evolution of the steady-state response in a more refined manner^25^.

Dynamics of the steady-state responses obtained after column-wise averaging of epochs mainly represent evolution of the oscillatory activity that is synchronized among the averaged EEG segments (those which survive the averaging). Therefore, a major issue regarding the feasibility of the methodology is the possible attenuation of response amplitudes due to variations in the phase of neural oscillations from one independent experimental run to another (i.e., among recordings). This topic needs to be addressed experimentally. However, evidence indicates that the phase of brain oscillatory responses is less variable than expected. In fact, several studies have reported a regularity in the expected phase of the human 80 Hz ASSR^47,48,49^. When latencies are estimated based on the phase of the oscillatory activity, the predictable effect of the intensity and the carrier frequency of the acoustic stimuli on the latency of the auditory responses has been observed (i.e., the latency decrease as the intensity and carrier frequency increase)^52,53,54^. Furthermore, typical maturational changes in amplitude and the left-to-right asymmetry in the hearing levels have been also observed when latencies are estimated from the phase of the ASSR^47,55,56,57,58^. When describing the time evolution of SSVEP using single-trial analysis, Labecki et al.^26^ observed that although inter-trial variability of the response amplitudes within the same subject was considerably high, variability of the phase was significantly less pronounced.

Based on their observations, Labecki et al.^26^ suggested that a minimum of 50 trials should be averaged to obtain a reliable estimation of the mean power envelope of the response. These results indicate that, even when the amplitude of the response is computed in single trials, averaging (of envelopes in that case) is needed to report trustworthy results. Moreover, the inter-trial variability in the amplitude of SSVEP reported by Labecki et al.^26^ suggests that the computation of this parameter in single trials can be highly influenced by background noise. Considering the evolution of the signal-to-noise ratio presented in Figure 2, the computation of the response in the averaged signal instead of single trials significantly reduces the number of EEG segments needed to be processed to obtain reliable measurements. Additionally, the low variability in phase obtained by Labecki et al.^26^ supports the idea that the column-wise averaging of epochs presented here is a valid procedure for computing the dynamics of oscillatory evoked potentials.

Averaging the data at different levels leads to different interpretation of the results. Regarding oscillatory evoked potentials, computing the response amplitude after the time-domain averaging of independent runs implies analyzing only time-locked oscillations (i.e., those that survive the averaging). This procedure may filter relevant information regarding the dynamics of the response in individual trials. However, it guaranties a sufficiently high signal-to-noise ratio of the measurements. This aspect might be of significance when the responses are close to the electrophysiological threshold, a condition in which the detection of the entrainment can be compromised due to low signal-to-noise ratio of the measurement.
